# Simulation Models of Leaf Area Index and Yield for Cotton Grown with Different Soil Conditioners

**DOI:** 10.1371/journal.pone.0141835

**Published:** 2015-11-04

**Authors:** Lijun Su, Quanjiu Wang, Chunxia Wang, Yuyang Shan

**Affiliations:** 1 School of Sciencem, Xi’an University of Technology, Xi’an, Shaanxi, China; 2 Institute of Water Resources and Hydro-electric Engineering, Xi’an University of Technology, Xi’an, Shaanxi, China; 3 Shihezi University, Shihezi, Xinjiang, China; Huazhong university of Science and Technology, CHINA

## Abstract

Simulation models of leaf area index (*LAI*) and yield for cotton can provide a theoretical foundation for predicting future variations in yield. This paper analyses the increase in *LAI* and the relationships between *LAI*, dry matter, and yield for cotton under three soil conditioners near Korla, Xinjiang, China. Dynamic changes in cotton *LAI* were evaluated using modified logistic, Gaussian, modified Gaussian, log normal, and cubic polynomial models. Universal models for simulating the relative leaf area index (*RLAI*) were established in which the application rate of soil conditioner was used to estimate the maximum *LAI* (*LAI*_m_). In addition, the relationships between *LAI*_m_ and dry matter mass, yield, and the harvest index were investigated, and a simulation model for yield is proposed. A feasibility analysis of the models indicated that the cubic polynomial and Gaussian models were less accurate than the other three models for simulating increases in *RLAI*. Despite significant differences in *LAI*s under the type and amount of soil conditioner applied, *LAI*_m_ could be described by aboveground dry matter using Michaelis-Menten kinetics. Moreover, the simulation model for cotton yield based on *LAI*_m_ and the harvest index presented in this work provided important theoretical insights for improving water use efficiency in cotton cultivation and for identifying optimal application rates of soil conditioners.

## Introduction

Soil salinisation and water shortages are important problems that restrict the efficiency of agricultural production in the south of Xinjiang, China [1, 2]. The use of soil conditioners is necessary to improve saline and alkaline land and to conserve irrigation water in this special arid climate. Cotton is second only to grain as the most important economic crop, especially in Xinjiang province, and its yield has reached 50 percent of the total yield for the entire country. Research on simulation models of cotton leaf area index (*LAI*) and yield under soil conditioners can provide a comprehensive treatment theory to improve production and to reform the saline-alkaline soil.

The application of various soil conditioners is widely practiced to improve the physical quality of coarse-textured soils. The effects of some soil conditioners on aggregate stability and pore-size distribution have been discussed by Asghari et al. [3]. Several studies on the application of natural organic soil conditioners [4–8] and synthetic organic soil conditioners [9–12] to sandy and sandy-loam soils have indicated an improvement in water content. Information on the quantitative effects of soil conditioners on the simulation of crop *LAI* and yield, however, is still inadequate.

An ideal method for simulating the development of *LAI* and crop yields should require a minimal amount of input data and be based on the underlying physiological and phenological processes that govern these properties in plants. Two approaches to this problem have been developed: estimation [13–19] and models of species growth [20]. Methods of estimation generally rely on determining the LAI by remote sensing [21,22] or by direct measurements [15]. Conversely, models of species growth, such as the logistic, Gompertz, Richards, or Chanter models, are always based on a theoretical foundation. In each case, one or more of the parameters in these models represents some physical characteristic. Such models describe how population sizes and biomass change over time, nutrition or water content. The logistic model, a classic population model of this type [23–26], has been widely used to simulate population growth and has demonstrated a good level of accuracy. Overman et al. [25, 27–30] modelled biomass production in forage grasses over time in a way that accounted for key environmental factors by extending the logistic model to incorporate the timing of harvest and water use efficiency in different years as parameters that were treated as functions of nutrition and water content. Yang et al. [31] incorporated environmental data to develop a dynamic revised logistic model based on fruit growth at different planting densities and seasons for describing the growth of individual tomatoes.

While the logistic model and its extended variants have usually simulated the stage of crop growth, they are not appropriate for modelling the late stable period or the period of decline. A few numerical models for simulating crop growth have been developed, including the modified logistic, cubic polynomial, and exponential growth (also known as Gaussian, modified Gaussian, and log normal) models [32]. These models have primarily been used to simulate growth in maize, wheat, and cotton [33–36].

Many attempts have been made to improve the accuracy and utility of the models of crop growth. To improve the general ability of models to simulate crop growth, some modellers have attempted to introduce flexibility so that simulation could be adjusted to agree with observation. Arkin et al.^[^ ^37^ ^]^ proposed the concept of a hybrid “spectral-physiological” model able to use Landsat data, which led to the SORGF model [38]. Later models included SOYGRO [39] and SORKAM [40]. Users of SOYGRO can adjust a parameter affecting photosynthetic rate to improve agreement between simulated and measured biomasses. In SORKAM, a parameter affecting the foliar expansion rate can be adjusted to equate simulated and measured *LAI*s. Barns et al. [41] modified CERES-Wheat [42] to allow the model to accept observed *LAIs* and to adjust related parameters as a function of *LAI*. These procedures objectively calibrate model response to actual field conditions for each application, but they require the same inputs that CERES-Wheat requires. Baez-Gonzalez et al. [43] recently modelled crop growth using satellite and field data to monitor and estimate corn yield on a large scale.

The objective of the present study was to develop and evaluate a method for simulating *LAI* and yield in cotton based on selected mathematical models and predefined soil conditioners. Four models of crop growth simulated changes in the *LAI* of cotton growing in the study area. The effects of various soil conditioners on maximum *LAI* (*LAI*_m_) and water-use efficiency (WUE) were also analysed. The maximum dry matter yield of cotton was estimated by *LAI*_m_ based on Michaelis-Menten kinetics. Finally, a mathematical model for simulating cotton yield based on *LAI*_m_ and the cotton harvest index (*HI*) was developed.

## Materials and Methods

There were no specific permissions required for these locations, because the experiments were carried out on private land, and the owner of the land gave permission to conduct the study. Because we researched on cotton growth and soil conditioners in saline-alkali land, the field studies did not involve endangered or protected species.

### Experimental field

The experiments were conducted at an irrigation experimental station in the town of Senior, 15 km south of Korla (41.68°N, 86.06°E, 950 m.a.s.l.), Xinjiang, China. This area is dry and rainless with a typical arid and semi-arid continental climate. The average annual rainfall is 53.5–62.9mm, and the average annual evaporation capacity is 2275-2789mm. The soil of the experimental plots is classified as sandy, in which the percentage compositions for sand, silt, and clay are 88.87%, 9.41%, and 1.72%, respectively. Soil bulk density within 100 cm is 1.63 g·cm^-3^, field capacity is 14.63% cm^3^·cm^-3^, and saturation moisture content is 19.46% cm^3^·cm^-3^. The average salinity content within 50 cm is 0.053%, and the groundwater table is 1.5 m [2].

### Experimental design

**Fig 1:**
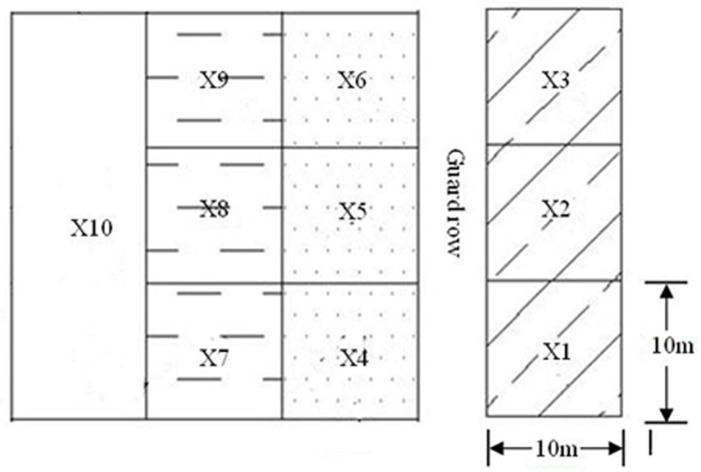
Arrangement of the treatments. X1: polyacrylamide, 0.75g·m^-2^; X2: polyacrylamide, 1.5g·m^-2^; X3: polyacrylamide, 3.0g·m^-2^; X4: fulvic acid, 75g·m^-2^; X5: fulvic acid, 150g·m^-2^; X6: fulvic acid, 150g·m^-2^; X7: gypsum, 199.5g·m^-2^; X8: gypsum, 400.5g·m^-2^; X9: gypsum, 501.0g·m^-2^; X10: CK, check treatment without soil conditioner.

Three types of soil conditioners were used in a random complete block design. The soil conditioners polyacrylamide (PAM), fulvic acid (FA) and gypsum were each tested at three application rates. A control treatment with no conditioner was included in the design. Table 1 shows the application rates of soil conditioners. The irrigation rate is 4.425 L·ha^-1^ and the irrigation period is 7 days for all experiments. The size and arrangement of the experimental plots are shown in Fig 1. Each treatment had three replicates. The plots were sown on 23 April, 2011 and irrigated 11 times between 17 June and 28 August.

**Table 1:** Experimental design for the soil conditioners.

Soil conditioner	Treatment	Application rate (g·m^-2^)
	X1	0.75
PAM	X2	1.5
	X3	3.0
	X4	75
FA	X5	150
	X6	300
	X7	199.5
Gypsum	X8	400.5
	X9	501.0
CK	X10	0

**Fig 2:**
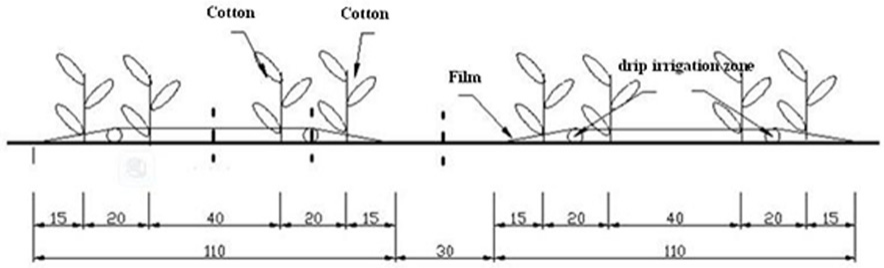
Cross section of the planting mode for the treatments of soil conditioners (distances in cm).

Drip irrigation was used, and each conditioner treatment had four rows of cotton plants and two lines of drip emitters under plastic film (Fig 2). The discharge rate was 3.2 L·h^-1^ for the drip irrigation zone, and the distance between emitters was 30 cm. The soil conditioners were uniformly mixed with the topsoil before tillage.

### Methods of measurement for indexes of cotton growth

Sample method: the best-growing and strongest seedlings were chosen for all treatments, and a total of six cotton plants from an inner and an outer row were randomly chosen for sampling from each treatment. Leaf area was calculated by multiplying the product of main-vein length and maximum width by the factor 0.7.

Dry matter method: the stems, leaves, buds, bolls, and main roots were collected from the six plants in each treatment. Dry matter mass was obtained by oven drying the samples to constant weight. In each treatment, all the cotton was collected from the unit areas (2 m ×2 m) and oven dried. The total dry matter mass was calculated by multiplying the mass of dried samples in the unit areas by the areas of the plots.

The data for cotton biomass were collected for three plants in each treatment. The masses of the cotton from the upper, lower, and middle branches were recorded separately. Measurements were thus collected from nine branches for each treatment. The data collected included the number of branches on each plant, the length of each branch studied, the number of leaves on each branch studied, the main-vein length and the maximum leaf width of each recorded leaf. Leaf area was calculated by multiplying the product of main-vein length and maximum width by the factor 0.7, and the arithmetic mean value of all leaf areas was used as the average leaf area. *LAI* was calculated as:

LAI(t)=(B(t)×I(t)×W(t)/10000)/S (1)

where *B* is the number of branches, *I* is the number of leaves, *W* is the average leaf area in cm^2^, *S* is the average area covered by a single cotton plant in m^2^, and *t* is the number of days from the beginning of the year (i.e. 1 January).

### Calculations of growing degree days (GDD)

The daily GDD was calculated as the difference between the daily average temperature (*T*_*avg*_) and the base temperature that crops need for growth (*T*_*base*_)^[^ ^44^ ^]^:

$$GDD=\sum_{i}(T_{{avg}_{i}}-T_{{base}_{i}})$$ (2)

*T*_*base*_ is a constant for specific areas and crops. The daily average temperature was calculated as the arithmetic mean of the daily maximum (*T*_*x*_) and minimum (*T*_*n*_) temperatures, subject to the limitations that it could not exceed *T*_*upper*_ (the temperature above which no further increase in the rate of plant growth is observed) or be less than *T*_*base*_. This method was developed by the Food and Agriculture Organization (FAO):

$$T_{avg}=\frac{\left(T_{x}^{*}+T_{n}^{*}\right)}{2}$$ (3)

$$\left\{ \begin{array}{l} T_{x}^{*}=T_{upper}\;if\;T_{x}^{*}\geq T_{upper}\\ T_{x}^{*}=T_{base}\;if\;T_{x}^{*}\leq T_{base}\\ T_{x}^{*}=T_{x}\;otherwise \end{array}\right.\;T_{n}^{*}=T_{upper}\;if\;T_{n}^{*}\geq T_{upper}$$

### Models of increasing LAI

The growth of crops is highly dependent on cumulative temperature over the growing season. The concept of GDD was thus proposed [44–46]. Even though growth areas and periods vary for different cotton crops, the GDDs needed during the entire growth and development period are generally the same. Thus, using GDD to characterise cotton growth can minimise the differences associated with different areas and years.

We used five mathematical models based on GDD to simulate the dynamic changes in *LAI* over the experimental period: the modified logistic, Gaussian, modified Gaussian, log normal, and cubic polynomial models. The cubic polynomial model is a simple empirical model. The other models are exponential models, some of which have parameters in common that correspond to specific physical variables.

The modified logistic model [31,47] is defined as:

$$LAI=\frac{LAI_{m}}{1+e^{a+b\cdot GDD+c\cdot GDD^{2}}}$$ (4)

where *a*, *b*, and *c* are undetermined coefficients. A GDD of 0 corresponds to 1 January.

The log normal model [32] is defined as: 00a

$$LAI=LAI_{m}\cdot \text{exp}\left[-0.5{\left(\frac{\text{ln}(GDD/a)}{b}\right)}^{2}\right]$$ (5)

where *a* represents the day on which *LAI* reaches its maximum and *b* is an undetermined coefficient. Eq 5 indicates that *LAI* = *LAI*_m_ when *GDD* = *a*.

The cubic polynomial model is defined as:

$$LAI=a_{0}+a_{1}\cdot GDD+a_{2}\cdot GDD^{2}+a_{3}\cdot GDD^{3}$$ (6)

where *a*_0_, *a*_1_, *a*_2_, and *a*_3_ are undetermined coefficients. The cubic polynomial model is thus only useful for simulating changes in *LAI* and cannot be extended to describe other variables of interest.

The Gaussian model (Eq 7 below) was first described by the German mathematician Gauss and takes the form of a curve whose single peak is determined by three parameters. The modified Gaussian model (Eq 8) is derived from the original Gaussian model[32].

$$LAI=LAI_{m}\cdot exp\left[-0.5{\left(\frac{GDD-a}{b}\right)}^{2}\right]$$ (7)

$$LAI=LAI_{m}\cdot \text{exp}\left[-0.5{\left(\frac{\left|GDD-a\right|}{b}\right)}^{c}\right]$$ (8)

where a, b and *c* re undetermined coefficients. *a* represents the GDD when *LAI* reaches its maximum. The parameters in the above models were fitted using the genetic algorithm of the MATLAB software package.

### Statistical analysis

The performance of these models was evaluated using three statistical tools: the root-mean-square error (RMSE), the coefficient of determination, and the relative error (Re). RMSE is a statistical tool for analyzing the deviation between measured and simulated values when testing models. Low RMSEs represent better simulated results. RMSE is defined as:

$$RMSE=\sqrt{\frac{\sum_{i-1}^{n}{\left(O_{i}-S_{i}\right)}^{2}}{n}}$$

where *O*_*i*_ is a measured value, *S*_*i*_ is a predicted value generated by the model, and *n* is sample size.

The coefficient of determination (*R* ^2^) is another tool for evaluating the magnitude of the difference between measured values and the predictions generated by a model. A large *R* ^2^ indicates a small deviation between the simulated and experimental data.

$$R^{2}=1-\frac{\sum_{i-1}^{n}{\left(O_{i}-S_{i}\right)}^{2}}{\sum_{i-1}^{n}{\left(O_{i}-{\bar{O}}_{i}\right)}^{2}}$$

Re is defined as:

$$Re=\sqrt{\frac{\sum {\left(u_{r}-u_{s}\right)}^{2}}{\sum u_{r}^{2}}}$$

where *u*_*r*_ represents measured values and *u*_*s*_ represents predicted values.

## Results

### LAI simulation models based on GDD

**Fig 3:**
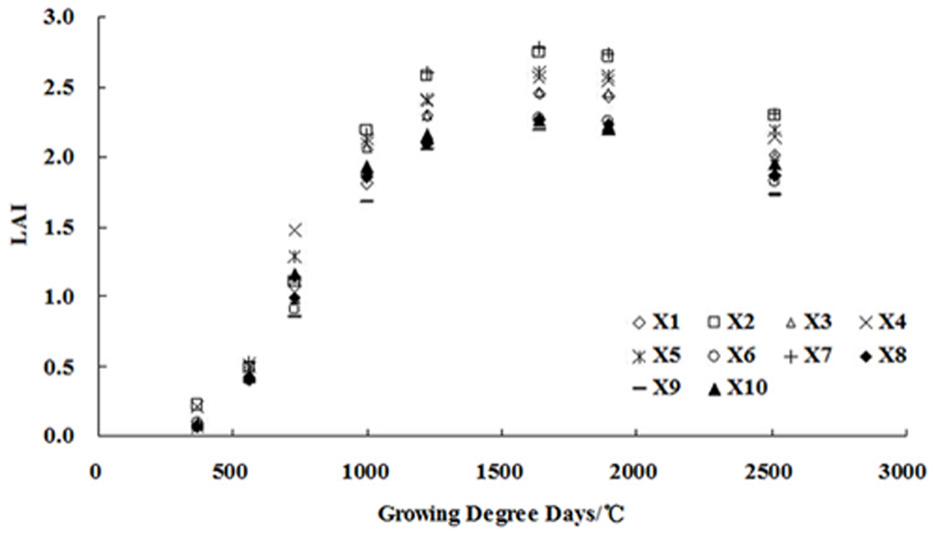
Dynamic changes in cotton *LAI* with GDD for the treatments. Even though the *LAI* in different treatments was not identical, the trends in *LAI* over *GDD* were similar for all treatments: *LAI* increased rapidly between 200°C and 1500°C, more gradually between 1500°C and 2000°C, and then decreased slowly.

The dynamic changes in the *LAI* of cotton grown over GDD for different treatments of soil conditioners are shown in Fig 3. *LAI* varied significantly amongst the treatments. The trend in *LAI* over the growing season, however, was similar for all treatments.

This similarity allowed the normalisation of the *LAI* data to simplify subsequent analyses. The data for X1 (PAM, 0.75 g·m^-2^), X2 (PAM, 1.5 g·m^-2^), X4 (FA, 75 g·m^-2^), X5 (FA, 150 g·m^-2^), X7 (Gypsum, 199.5 g·m^-2^), and X8 (Gypsum, 400.5 g·m^-2^) were subsequently used to analyse and build models, which were tested with the data from the other treatments using the relative *LAI* (*RLAI*), defined as:

$$RLAI=\frac{LAI}{LAI_{m}}$$ (9)

The results of these calculations for treatments X1, X2, X4, X5, X7, and X8 are shown in Table 2. The standard deviations of the *RLAI*s ranged from 0 to 0.0308 for the complete growing season. The general trends in the change of *RLAI* for the six treatments were similar. The impact of the different soil conditioners and their application rates on changes in *LAI* can consequently be ignored, and the unified, normalised growth models for *LAI* should use the mean values of *RLAI* based on Eq 9.

**Table 2:** *RLAI* for six treatments at different times and the mean *RLAI*s for all treatments.

GDD (°C)	*RLAI*						Mean *RLAI*	Standard deviation
	X1	X2	X4	X5	X7	X8		
368.5	0.03	0.04	0.04	0.03	0.03	0.03	0.03	0.0052
558.2	0.16	0.18	0.19	0.20	0.19	0.19	0.19	0.0138
725.4	0.44	0.40	0.46	0.46	0.41	0.44	0.43	0.0251
996.4	0.74	0.80	0.81	0.82	0.78	0.82	0.79	0.0308
1221.7	0.93	0.94	0.93	0.93	0.94	0.92	0.93	0.0075
1639.0	1.00	1.00	1.00	1.00	1.00	1.00	1.00	0
1894.1	0.97	0.97	0.99	0.99	0.98	0.99	0.99	0.0098
2513.6	0.82	0.84	0.83	0.84	0.83	0.83	0.83	0.0071

**Fig 4:**
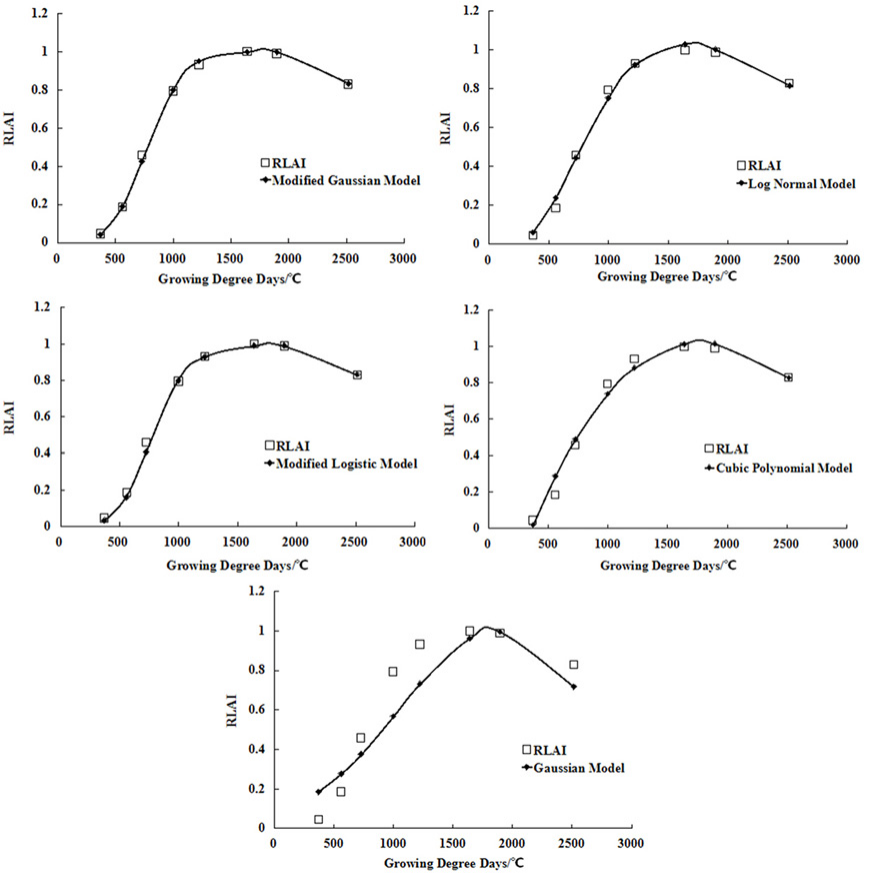
Comparisons of the measured and simulated Mean *RLAI* values for the five models. (a) Mean *RLAI* values were fitted by modified Gaussian model. (b) Mean *RLAI* values were fitted by log normal model. (c) Mean *RLAI* values were fitted by modified logistic model. (d) Mean *RLAI* values were fitted by cubic polynomial model. (e) Mean *RLAI* values were fitted by Gaussian model.

The mean *RLAI*s for the above six treatments were simulated using the modified logistic, Gaussian, modified Gaussian, log normal, and cubic polynomial models based on GDD. In each case, the model’s parameters were fitted using the genetic algorithm of MATLAB. The simulated results of the different models are shown in Fig 4. All models agreed well with the observed changes in *LAI*, especially for the late stable period and the period of decline. The correlation coefficients for the relationship between model output and experimental data were above 0.98 for all models except the Gaussian model.

Table 3 shows the fitted parameters and simulation errors for the five models. Except for the Gaussian model, the models produced high correlation coefficients, low Re percentages, and predictions that agreed well with the experimental data for the late stable period and the declining period of crop growth. The modified Gaussian model was the most accurate of those tested.

**Table 3:** Fitted parameter values and simulation errors for the five models.

Model	Expression	Re (%)	*R* ^*2*^	RMSE	Parameter number
Modified Gaussian Model	$RLAI=0.9968\;\text{exp}\left[-0.5{\left(\frac{\left|CGDD-1772.9780\right|}{928.9753}\right)}^{4.4947}\right]$	0.95	0.9996	0.0067	4
Log Normal Model	$RLAI=1.0298exp\left[-0.5{\left(\frac{ln(CGDD/1638.4502)}{0.6294}\right)}^{2}\right]$	3.84	0.9939	0.0272	3
Modified Logistic Model	$RLAI=\frac{1.03}{1+e^{7.2955-0.01191\cdot CGDD+3.3565\times 10^{-6}\cdot CGDD^{2}}}$	1.66	0.9989	0.0117	4
Cubic Polynomial Model	$\begin{array}{l} RLAI=6.8419\times 10^{-11}CGDD^{3}-7.7320\times 10^{-7}CGDD^{2}\\ \;+2.1006\times 10^{-3}CGDD-0.6533 \end{array}$	5.06	0.9894	0.0358	3
Gaussian Model	$RLAI=1.1186\text{exp}\left[-0.5{\left(\frac{CGDD-1856.3693}{810.3980}\right)}^{2}\right]$	15.7	0.8972	0.1113	4

The modified Gaussian, log normal, and Gaussian models are all based on exponential functions. Parameter *a* in these models represents the maximum *RLAI* (*RLAI*_max_ = 1). The deviations between the value of *a* in the three models and the experimental *RLAI*_max_ were -0.0032 for the modified Gaussian, 0.0298 for the log normal, and 0.1186 for the Gaussian models. That is, the *RLAI*_max_ predicted by the modified Gaussian model was lower than the measured value, while those predicted by the log normal and Gaussian models were higher than the measured values. The absolute magnitude of the deviation between *a* and *RLAI*_max_, however, determines the predictive accuracy of the model, and the modified Gaussian model performed best for this criterion.

The flexibility and general applicability of a model are both highly sensitive to the number of its parameters. Fewer parameters can improve the general applicability of a model but may also decrease the accuracy of its predictions in any given case. The log normal and Gaussian models both have three parameters (Table 3), while the modified logistic, modified Gaussian, and cubic polynomial models have four parameters. The larger number of parameters in the latter group renders them more complex. The log normal model may thus be preferable for simulating dynamic changes in *RLAI* when high precision is not required.

### Relationship between soil conditioner and LAI

**Fig 5:**
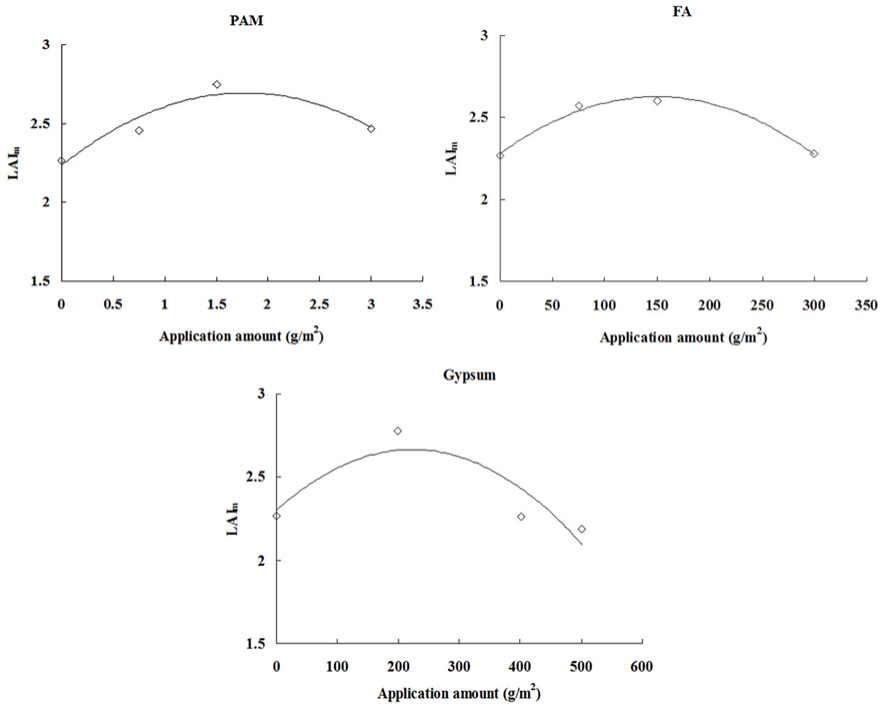
Relationship between application rate and *LAI*_m._ When application amount = 0 g·m^-2^, it was the *LAI*_*m*_ value of CK treatment.

The above analysis indicated that the type and application rate of soil conditioner had no effect on the trend of *LAI* over the growing season but had some effect on *LAI*_m_. Fig 5 shows the measured values of *LAI*_m_ at the different application rates for the three soil conditioners.

*LAI*_m_ was most influenced by the soil conditioner, with the same irrigation system. The optimal application rates for the soil conditioners were: PAM ≈ 0.75 g·m^-2^–3 g·m^-2^, FA ≈ 75 g·m^-2^–300 g·m^-2^, and gypsum ≈ 0 g·m^-2^–400.5 g·m^-2^. Each soil conditioner was tested at three rates of application, so the relationship between application rate and *LAI*_m_ was fitted by the simple quadratic polynomial as:

$$\text{PAM}:\;LAI_{m}=-0.1463\cdot I^{2}+0.5196\cdot I+2.2341,\;{\text{R}}^{2}=0.89**$$ (10)

$$\text{FA}:\;LAI_{m}=-1.5522\times 10^{-5}\cdot I^{2}+4.6446\times 10^{-3}\cdot I+2.2781,\;{\text{R}}^{2}=0.98**$$ (11)

$$\text{Gypsum}:\;LAI_{m}=-7.3212\times 10^{-6}\cdot I^{2}+3.2596\times 10^{-3}\cdot I+2.3018,\;{\text{R}}^{2}=0.76**$$ (12)

where *I* is the rate of application in g·m^-2^. The first order derivatives of Eqs 10–12 are solved, and let

$$\frac{dLAI_{m}}{dI}=0$$

The optimal application rate of soil conditioner can then be calculated for obtaining the best *LAI*_m_, as shown in Table 4.

**Table 4:** Optimal application rate and *LAI*_m_ for the three soil conditioners.

Soil conditioner	Optimal application rate (g·m^-2^)	*LAI*_m_
PAM	1.78	2.70
FA	149.6	2.63
Gypsum	222.6	2.66

### Relationship between LAI and dry matter mass

**Fig 6:**
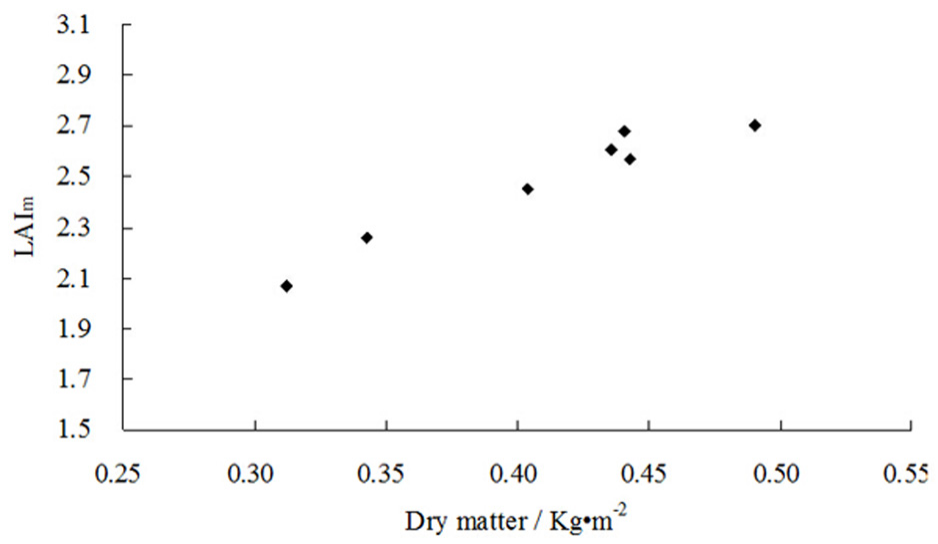
Relationship between LAI and dry matter mass.

The aboveground dry biomass of plants will generally determine the *LAI*, because biomass dictates the amount of light that can be absorbed and thus the rate of dry-matter production. Differences in ecological factors between study locations and growth periods will affect the rate at which dry mass increases. Increases in dry-matter productivity, however, will lead to higher *LAI*s in any given growth period. Fig 6 shows the relationship between *LAI*_m_ and the measured relative dry biomass. The data in Fig 6 were obtained from treatments X1, X2, X4, X5, X7, X8, and X10.

*LAI* correlated strongly with aboveground dry biomass, which followed Michaelis-Menten kinetics [48]. The rate of increase in *LAI* thus decreases as the amount of dry mass increases. To facilitate subsequent analyses, we used the experimental data on dry matter and *LAI* in each growth period to analyse the relationship between these two quantities:

$$LAI=\frac{P\cdot M}{1+Q\cdot M}$$ (13)

where *M* is the aboveground dry biomass, *P* and *Q* are undetermined coefficients. To calculate the undetermined coefficients *P* and *Q*, Eq 13 is linearised:

$$\frac{1}{LAI}=\frac{1}{P\cdot M}+\frac{Q}{P}$$ (14)

The linear relationship between *LAI*(*t*)^-1^ and *M* ^-1^ was analysed by the least squares method, and the undetermined coefficients *P* and *Q* were obtained by parameter transformation. The relationship shown in Fig 6 is defined by:

$$LAI=\frac{10.1317\cdot M}{1+1.6312\cdot M}$$ (15)

Here, the sample number is 10, the correlation coefficient is 0.9557, Re is 1.83%, and RMSE is 0.0456. The difference between the measured and calculated values was not significant (p > 0.05). The relationship between dry biomass and *LAI* was defined as:

$$M=\frac{LAI}{P-Q\cdot LAI}=\frac{LAI}{10.1317-1.6312\cdot LAI}$$ (16)

### A mathematical model for predicting yields

The harvested portion of this biomass (yield) is reflected in the harvest index (*HI*):

Y=B⋅HI (17)

Once the total biomass (*B*) has been determined, the crop yield (*Y*) can be calculated as the product of *B* and *HI* (Eq 17). We partitioned the total biomass into two parts: dry matter and yield:

$$B=M_{m}+Y$$ (18)

where *M*_*m*_ is the peak dry mass produced. Based on Eqs 17 and 18, the yield can be predicted as:

$$Y=\frac{HI}{1-HI}\cdot M_{m}$$ (19)

From Eq 16, the relationship between *M*_*m*_ and *LAI*_m_ can be defined as:

$$M_{m}=\frac{LAI_{m}}{P-Q\cdot LAI_{m}}$$ (20)

Here, *P* and *Q* are the parameters in Eq 16.

Table 5 shows the yield, dry biomass, total biomass, and *HI* for the treatments at the end of the cotton wadding stage. *HI* was calculated by Eqs 17 and 18. Both conditioner type and application rate affected cotton yield and biomass. Moreover, the yields were maximal at the intermediate application rates of the three conditioners, i.e. in treatments X2 (PAM, 1.5 g·m^-2^), X5 (FA, 150 g·m^-2^), and X7 (Gypsum, 199.5 g·m^-2^). The optimal application rates for the three soil conditioners at the same level of irrigation were: PAM ≈ 0.75 g·m^-2^–3 g·m^-2^, FA ≈ 75 g·m^-2^–300 g·m^-2^, and gypsum ≈ 0 g·m^-2^–400.5 g·m^-2^. Both yield and biomass varied among treatments, but the *HI*s were similar, ranging from 0.5012 to 0.5337. The mean *HI* for these treatments was 0.5148, and the standard deviation was 0.0115.

**Table 5:** Yield, dry biomass, total biomass, and *HI* for each treatment.

	Yield (t·ha^-1^)	Dry biomass (t·ha^-1^)	Total biomass (t·ha^-1^)	HI
X1	4.4400	4.0400	8.4800	0.5236
X2	4.6500	4.6012	9.2512	0.5026
X3	4.1700	4.1503	8.3203	0.5012
X4	4.5225	4.4306	8.9531	0.5051
X5	4.7550	4.3552	9.1102	0.5219
X6	3.9675	3.8347	7.8022	0.5085
X7	4.935	4.4054	9.3404	0.5284
X8	3.9225	3.4272	7.3497	0.5337
X9	3.8550	3.6157	7.4707	0.5160
X10	3.2100	3.1220	6.3320	0.5070

Combining Eqs 16, 19 and 20 and setting *HI* at 0.5148, the cotton yield predicated by the model can be obtained as:

$$Y=\frac{HI}{1-HI}\cdot M_{m}=\frac{0.5148}{1-0.5148}\cdot \frac{LAI_{m}}{10.1317-1.6312\cdot LAI_{m}}=\frac{0.5148\cdot LAI_{m}}{4.9159-0.7915\cdot LAI_{m}}$$ (21)

### Feasibility analysis of the models

The data from treatments X3 (PAM, 3 g·m^-2^), X6 (FA, 300 g·m^-2^), X9 (Gypsum, 501 g·m^-2^), and X10 (CK) were used to test and verify the accuracy and feasibility of the models. *LAI*, *LAI*_m_, and yield predicted by the proposed models were compared to the measured values. Accuracy and feasibility were also evaluated by error analysis.

Universal models for calculating *LAI* can be established by combining the models described in Table 3 with Eq 9. For example, *LAI* calculated with the modified logistic model would be:

$$LAI=LAI_{m}\cdot RLAI=\frac{1.03\cdot LAI_{m}}{1+e^{7.2955-0.01191\cdot GDD+3.3565\times 10^{-6}\cdot GDD^{2}}}$$ (22)

**Fig 7:**
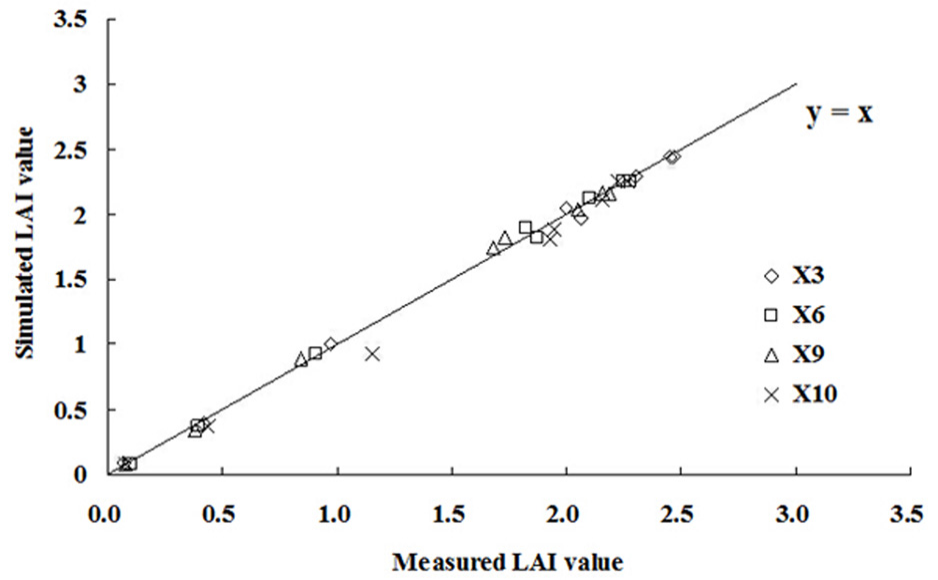
Predicted *LAI*s calculated with Eq 22.

Fig 7 shows the simulated *LAI*s for the tested treatments calculated by Eq 22. The simulated error analysis for the *RLAI* of cotton grown under the different soil conditioners for each of the developed universal models is shown in Table 6.

**Table 6:** *LAI*s predicted by the various universal models.

		Modified Logistic Model	Modified Gaussian Model	Log Normal Model	Cubic Polynomial Model	Gaussian Model
	*R* ^*2*^	0.9978	0.9967	0.9847	0.9658	0.8816
X3	Re (%)	2.32	2.84	6.07	9.08	16.89
	RMSE	0.0379	0.0464	0.0993	0.1484	0.2763
	*R* ^*2*^	0.9978	0.9967	0.9874	0.9682	0.8888
X6	Re (%)	2.28	2.79	5.44	8.64	16.17
	RMSE	0.0342	0.0418	0.0816	0.1296	0.2425
	*R* ^*2*^	0.9965	0.9956	0.9897	0.9702	0.8997
X9	Re (%)	2.89	3.25	4.97	8.47	15.55
	RMSE	0.0412	0.0463	0.0709	0.1207	0.2217
	*R* ^*2*^	0.9949	0.9956	0.9890	0.9776	0.8819
X10	Re (%)	3.39	3.15	5.00	7.13	16.37
	RMSE	0.0554	0.0515	0.0818	0.1167	0.2679

The universal models can predict how *LAI* will change with GDD. *LAI*_m_ thus has a significant impact on the predicted *LAI*, and the accuracy with which it is estimated directly determines the accuracy of the predicted *LAI* obtained with the models. From the results presented in Table 6, we can draw the following conclusions: (1) for most treatments, the Gaussian and cubic polynomial models had higher RMSE and Re values than did the other three models, and (2) the modified logistic, modified Gaussian, and log normal models had the highest correlation coefficients (*R* ^*2*^) for the relationship between predicted and experimental results, and the Gaussian model had the lowest. These results suggest that the modified logistic, modified Gaussian, or log normal models should be used to predict *LAI* for cotton grown in the study area.

The dry matter masses and yields achieved using the tested soil conditioners can be predicted with Eqs 16 and 21. The predicted results for X3, X6, and X9 are shown in Table 7. The deviations of the simulated values from the experimental data ranged from -2.840% to 0.460% for dry matter mass and from -4.570% to 3.292%.

**Table 7:** Simulated and measured dry matter mass and yields for X3, X6, and X9 and the deviations of the simulated values from the experimental data as percentages of the experimental values.

Treatment	*LAI*_m_	Dry matter mass (Kg·m^-2^)			Yield (Kg·m^-2^)		
		Observed	Simulated	Deviation (%)	Observed	Simulated	Deviation (%)
X3	2.47	0.415	0.404	-2.840	0.417	0.431	3.292
X6	2.28	0.353	0.355	0.460	0.397	0.379	-4.570
X9	2.29	0.362	0.358	-1.036	0.3855	0.382	-0.822

The results in Table 7 show that when *HI* is known, models of yield that rely on only a single parameter—*LAI*_m_—can be used to estimate cotton dry matter mass and yields with an acceptable level of accuracy. According to the relationship between *LAI*_m_ and the application rate of soil conditioners, *LAI*_m_ can be calculated from application rate by quadratic polynomial functions.

## Discussion

Normalising the measured *LAI* allowed us to disregard the impacts of the different soil conditioners and application rates on the dynamic changes in the *LAI* of cotton. We established universal models for the increase in *RLAI* based on the modified logistic, modified Gaussian, log normal, Gaussian, and cubic polynomial models. These models all accurately simulated the measured changes in *LAI* for cotton over the course of the growing season. The Gaussian and cubic polynomial models, though, were less accurate than the other three models.

**Fig 8:**
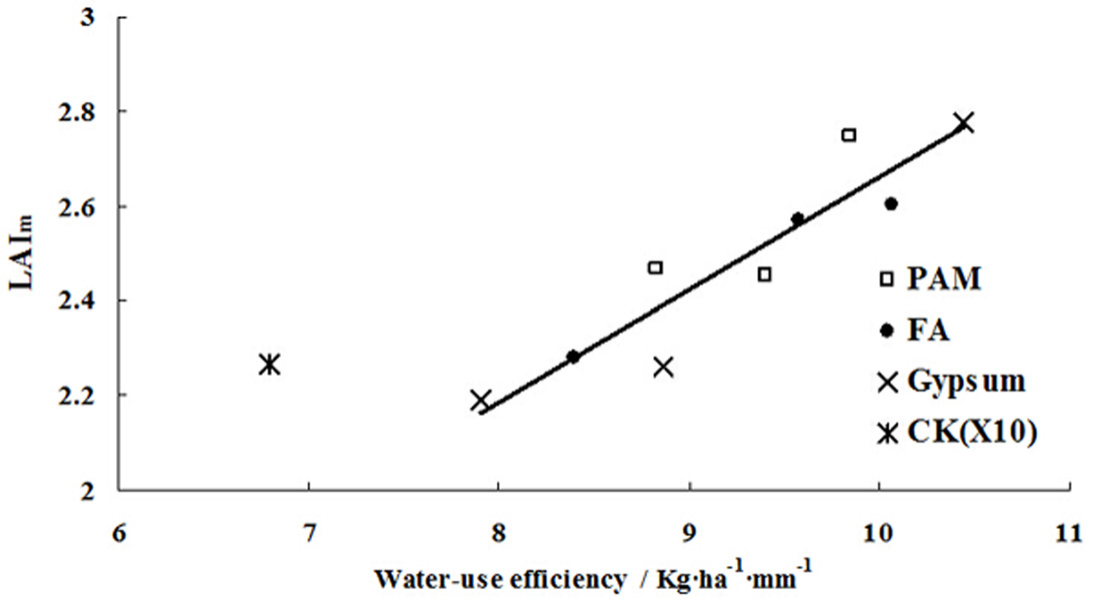
Relationship between transpiration and *LAI*_*m*_ for different soil conditioner treatments. Water-use efficiency (Kg·ha^-1^·mm^-1^) was calculated by dividing the cotton yield (Kg/ha) by the water consumption (mm). CK(X10) was the check treatment without soil conditioner.

The increase in *LAI* could be calculated by combining the *RLAI* growth model and *LAI*_m_. *LAI*_m_, though, was influenced by soil conditioner and application rate. The relationship between application rate and *LAI*_m_ was described by simple quadratic polynomial functions for each soil conditioner. Moreover, optimal application rates were obtained. Application rate, however, did not directly influence *LAI*_m_. Application rate has a direct impact on the capacity of soil to retain water, which can provide more soil water for transpiration and can limit evaporation. Transpiration is thus a core factor affecting *LAI*_m_ [49]. The relationship between water-use efficiency (WUE) and *LAI*_m_ was shown in the Fig 8.

Because the irrigation rate (4.425 L·ha^-1^) and the irrigation period (7 days) were more effective irrigation schedules for cotton growth in Korla, it can be seen from Fig 8 that *LAI*_*m*_ was linearly related WUE (*LAI*_*m*_ = 0.2383WUE + 0.2774, R^2^ = 0.86**) under the same irrigation schedule. However, WUE in CK(X10) was smaller than that in the other treatments under the same *LAI*_*m*_ value level, this is due to the water used to dry matter accumulation was more than that used to yield accumulation.

**Fig 9:**
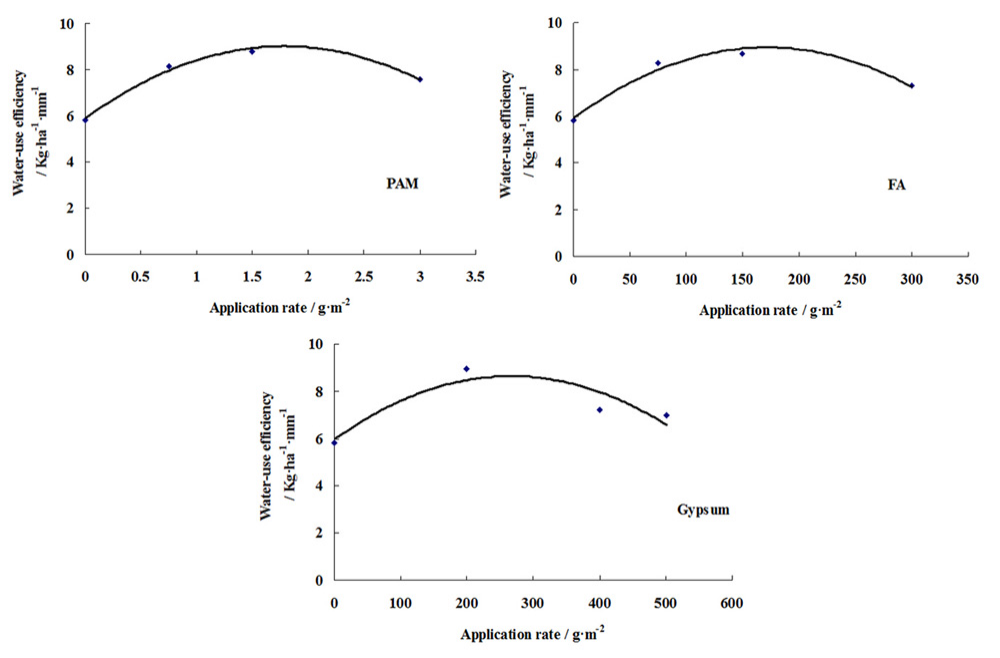
Relationship between application rate and water-use efficiency. When application rate = 0 g·m^-2^, it was the *WUE* value of CK treatment.

As expected, the soil conditioner and application rate were related to not only *LAI*, but also water-use efficiency (Fig 9). As shown in Fig 9, WUE can be described using a quadratic polynomial function of the application rate as:

$$\text{PAM}:\;WUE=-0.9799\cdot I^{2}+3.4987\cdot I+5.8941,\;{\text{R}}^{2}=0.98**$$ (23)

$$\text{FA}:\;WUE=-1.0229\times 10^{-4}\cdot I^{2}+3.5089\times 10^{-2}\cdot I+5.9337,\;{\text{R}}^{2}=0.96**$$ (24)

$$\text{Gypsum}:\;WUE=-3.7585\times 10^{-5}\cdot I^{2}+2.0064\times 10^{-2}\cdot I+5.9689,\;{\text{R}}^{2}=0.81**$$ (25)

where *I* is the rate of application in g·m^-2^. For the treatments of different soil conditioners, the water-use efficiency are higher than that in the control treatment without any soil conditioner. The peak WUE of three soil conditioners treatments were observed at 1.79g·m^-2^, 171.5g·m^-2^, 266.9g·m^-2^ for PAM, FA and Gypsum, respectively. Meanwhile, the optimal yields were observed at 1.5g·m^-2^ for PAM, 150g·m^-2^ for FA, and 199.5g·m^-2^ for Gypsum from Table 1 and Table 5. Thus, the more precise value ranges of optimal application rates for the three soil conditioners at the same level of irrigation were: PAM ≈ 1.5 g·m^-2^–1.79 g·m^-2^, FA ≈ 150 g·m^-2^–171.5 g·m^-2^, and gypsum ≈ 199.5 g·m^-2^–266.9 g·m^-2^.

The analysis of the relationship between *LAI*_m_ and dry matter found that *LAI*_m_ could be described by dry matter based on Michaelis-Menten kinetics, despite the large variation in *LAI*_m_ in the different treatments. Moreover, the dry matter mass of cotton could be calculated by *LAI*_m_ based on the model proposed in this paper. A simulation model for yield was established and the relationship between yield, dry matter mass, and *HI*. This model could use *LAI*_m_ to predict cotton yield.

The feasibility of the models presented in this paper was analysed by comparing simulated to observed data. The analysis indicated that the modified logistic, modified Gaussian, or log normal models were best for predicting dynamic changes in *RLAI* for cotton, and the log normal model was better when high precision was not required. Models that rely on a single parameter—*LAI*_m_—can estimate cotton dry matter mass and yields with an acceptable level of accuracy when HI is known.

## Supporting Information

S1 File Data used for building mathematical model and analyzing the feasibility of the models. (XLS)
